# Hepatic stellate cells specific liposomes with the Toll‐like receptor 4 shRNA attenuates liver fibrosis

**DOI:** 10.1111/jcmm.16209

**Published:** 2020-12-18

**Authors:** Yuwei Zhang, Yang Li, Tong Mu, Nanwei Tong, Ping Cheng

**Affiliations:** ^1^ Division of Endocrinology and Metabolism State Key Laboratory of Biotherapy West China Hospital Sichuan University, and Collaborative Innovation Center for Biotherapy Chengdu China; ^2^ State Key Laboratory of Biotherapy and Cancer Center West China Hospital Sichuan University, and Collaborative Innovation Center for Biotherapy Chengdu China

**Keywords:** hepatic stellate cells, liver fibrosis, reactive oxygen species, RNA interference, Toll‐like receptor 4

## Abstract

The hepatic stellate cells (HSCs) play a significant role in the onset of liver fibrosis, which can be treated by the inhibition and reversal of HSC activation. The RNA interference‐mediated TLR4 gene silencing might be a potential therapeutic approach for liver fibrosis. The crucial challenge in this method is the absence of an efficient delivery system for the RNAi introduction in the target cells. HSCs have an enhanced capacity of vitamin A intake as they contain retinoic acid receptors (RARs). In the current study, we developed cationic liposomes modified with vitamin A to improve the specificity of delivery vehicles for HSCs. The outcome of this study revealed that the VitA‐coupled cationic liposomes delivered the TLR4 shRNA to aHSCs more efficiently, as compared to the uncoupled cationic liposomes, both in the in vitro and in vivo conditions. Besides, as evident from the outcome of this study, the TLR4 gene silencing inhibited the HSCs activation and attenuated the liver fibrosis via the NF‐κB transcriptional inactivation, pro‐inflammatory cytokines secretion and reactive oxygen species (ROS) synthesis. Thus, the VitA‐coupled liposomes encapsulated with the TLR4‐shRNA might prove as an efficient therapeutic agent for liver fibrosis.

## 1 INTRODUCTION

Liver fibrosis is a dynamic and potentially reversible wound‐healing process that maintains liver integrity and signifies an early stage of liver cirrhosis.^1^ The aetiological factors of liver fibrosis include infections (hepatitis virus and schistosome), fatty liver disease (alcoholic and non‐alcoholic), cholestasis and autoimmune liver disorders.^2^ The typical pathological characteristic of liver fibrosis includes inflammatory cell infiltration and fibrogenesis, which occurs because of chronic liver injury.^3^

The hepatic stellate cells (HSCs), which cause liver fibrosis, are located in the sinus space between the sinusoidal endothelial cells (SEC) and the hepatocytes. HSCs are quiescent in the healthy liver and involved in vitamin A storage.^4^ However, HSCs are activated (aHSCs) in the damaged liver and differentiate into fibroblasts as well as myofibroblasts. HSCs activation is caused by the cytokines, growth factors, reactive oxygen species (ROS) and lipid peroxides, which are secreted by the cells adjacent to the liver injury site.^5^ The activated HSCs secrete a milieu of extracellular matrix (ECM) inflammatory factors, which promote fibrogenesis.^6^ Thus, HSCs inhibition and reversal of activation is a practical approach towards the development of liver fibrosis therapeutics.

Toll‐like receptors (TLRs) are a type of pattern‐recognition receptors and a crucial part of the innate immune system, which participates in endogenous danger signal detection.^7^ The pathogenic microbes, and injured/dying cells, express the highly conserved structural motifs PAMPs (pathogen‐associated microbial patterns), and DAMPs (damage‐associated molecular patterns), respectively, which are recognized by TLRs.^8^ Interestingly, recent findings suggest that TLR4 plays a crucial role in liver fibrosis progression, and inhibition of the TLR4 signalling pathway alleviates liver injury, which in turn attenuates liver fibrosis.^9^, ^10^ HSCs show the presence of intact TLR4 on their cell surface.^11^ The stimulation of these TLR4 induces the downstream signalling, which leads to the HSCs activation.^12^ Thus, HSCs associated with TLR4 might be an ideal therapeutic target for liver fibrosis.

RNA interference (RNAi) is an effective strategy to manipulate the pathogenic genes by the complementary base‐pairing mechanism.^13^ The RNAi is widely used in gene therapy against various disorders and delivered as a dsRNA molecule: small interfering RNAs (siRNAs), small hairpin RNAs (shRNAs), microRNA (miRNA) mimics or dicer substrate RNAs (dsiRNAs).^14^ As the TLR4 mediates inflammatory and pro‐fibrogenic signalling in HSC, the development of safe and efficient shRNA delivery vehicles targeted against the TLR4 to mitigate their signalling might serve as an efficient therapeutic agent for liver fibrosis.

In the present study, we encapsulated the plasmids expressing shRNA in VitA‐coupled liposomes to increase its specificity for HSCs, because HSCs have a significant ability of vitamin A (VitA) intake through retinoic acid receptors (RARs).^15^ We have also explored the effect of the TLR4 gene‐specific shRNA as a therapeutic agent in carbon tetrachloride (CCl4) induced liver fibrosis in mice.

## 2 MATERIALS AND METHODS

### 2.1 Isolation of primary HSCs, hepatocytes, Kupffer cells and liver sinusoidal endothelial cells (LSECs) from mouse liver

The primary murine HSCs and hepatocytes were isolated from mouse liver by using enzymatic digestion and density gradient centrifugation, as described previously.^16^ The primary Kupffer cells and LSECs were isolated from mouse liver using liberase‐based perfusion technique, low‐speed centrifugation and magnetic‐activated cell sorting (MACS).^17^ Isolated primary cells were cultured in the DMEM medium supplemented with 10% foetal bovine serum (FBS) and incubated at 37°C in a humidified atmosphere with 5% CO_2_.

### 2.2 Preparation of plasmid constructs expressing TLR4 specific shRNA

Four candidate shRNA sequences to specifically target the mouse TLR‐4 gene were designed with an online shRNA design tool. Additionally, a non‐specific shRNA sequence was used as the control. The sequences of the TLR4‐specific shRNA and random shRNA have been described previously.^18^ These oligonucleotides were annealed to form the double‐stranded DNA fragments, which were cloned into the RNAi pGPU6/neo vector. Large‐scale vectors were purified using an Endo‐free Plasmid Giga kit (Qiagen). We also measured the bacterial endotoxin in purified plasmid DNA by the Tachypleus Amebocyte Lysate (TAL) test.

### 2.3 Preparation of the VitA‐coupled liposomes and encapsulation of the TLR4‐shRNA plasmids

We encapsulated the TLR4‐shRNA plasmids in VitA‐coupled liposomes, as mentioned in previous studies.^15^, ^19^ Briefly, dioleoylphosphatidylethanolamine (DOPE), *O*,*O*′‐ditetradecanoyl‐*N*‐(α‐trimethylammonioacetyl) diethanolamine chloride (DC‐6‐14) and cholesterol were taken at a molar ratio of 3:4:3, and cationic liposomes were synthesized by the freeze‐dried empty liposomes (FDEL) method. Subsequently, 100 nmol of hydrated cationic liposomes and 200 nmol of vitamin A were mixed by vortexing at 25°C to prepare the VitA‐coupled liposomes. The weight ratio of 5:1 of the VitA‐coupled liposomes to the TLR4‐specific shRNA or control shRNA plasmid was maintained for encapsulation of shRNA in the VitA‐coupled liposomes.

### 2.4 Transfection efficiency assay in vitro

Approximately 5 × 10^4^ primary murine HSCs, hepatocytes, Kupffer cells or LSECs were seeded in each well of 24‐well plates and incubated overnight at 37°C and in humidified air with 5% CO_2_. Later, the cells were transfected with either 0.4 μg of pGPU6‐GFP encapsulated in 2 μg liposome or VitA‐coupled liposome. The percentage of the GFP‐positive cells was analysed using an inverted fluorescent microscope (Zeiss, Axiovert 200) after 72 hours of transfection.

### 2.5 Interference efficiency assay in vitro

The interference efficiency of the TLR4 in the HSCs, hepatocytes, Kupffer cells or LSECs was detected by quantitative real‐time PCR (qRT‐PCR). Concisely, 2 × 10^5^ primary murine HSCs were seeded in each well of the 6‐well plate and incubated overnight at 37°C in a humidified atmosphere with 5% CO_2_. Later, these cells were transfected with 2 μg TLR4 shRNA/10 μg VitA‐coupled liposomes or 2 μg random shRNA/10 μg VitA‐coupled liposomes. The cells were harvested, and total RNA was extracted using Trizol reagent after 72 hours. The mRNA level of the TLR4 was analysed by qRT‐PCR.

### 2.6 Animals

We procured the 6‐8 weeks old syngeneic female C57/BL6 mice from Hua Fu Kang Biological Technology Company and housed them in the animal research facility of Sichuan University. This study was ethically approved by the Institute's Animal Care and Use Committee.

### 2.7 Establishment of liver fibrosis in mice

Liver fibrosis in mice was induced, as mentioned previously.^20^ In brief, female C57BL/6J mice were injected intraperitoneally (i.p) with 100 μL of 20% carbon tetrachloride (CCl4) suspended in corn oil or plain corn oil as a control, twice a week for consecutive 6 weeks.

### 2.8 In vivo distribution and pharmacokinetics of the VitA‐lip‐TLR4‐shRNA

The organ distribution of the VitA‐lip‐TLR4‐shRNA in mice was detected as described previously.^15^, ^21^ Briefly, the VitA‐lip‐TLR4‐shRNA complex was radiolabeled with the [^3^H]retinol. The procedure adopted for [^3^H]retinol incorporation into liposomes was similar to that of VitA‐coupled liposomes preparation. The TLR4‐shRNA plasmids were encapsulated with [^3^H]retinol‐radiolabeled VitA‐coupled liposomes. Later, we injected the 100 μCi [^3^H]VitA‐lip‐TLR4‐shRNA complex (18 μg) either in the normal mice or in the CCl4‐induced hepatic fibrosis mice (n = 4 per group) via the tail vein injection. The blood samples were collected via tail vein nick over a 24 hours period. These mice were killed, and their organs were harvested 24 hours post‐treatment. The radioactivity in the blood and other organs was detected by liquid scintillation spectrometer (PerkinElmer).

### 2.9 Treatment of the CCl4‐induced liver fibrosis mice with the VitA‐lip‐TLR4‐shRNA

After 43 days of the CCl4 intraperitoneal injection (i.p), thirty mice were randomly divided into three groups, and each group contained ten mice. These mice were injected with normal saline (NS), 18 μg VitA‐lip‐random shRNA, 18 μg lip‐TLR4‐shRNA and 18 μg VitA‐lip‐TLR4‐shRNA, respectively, every other day for 10 times by intravenous tail injection. The mice were administered CCl4 twice a week (100 μL) until the last liposome complex treatment administration. Six out of the ten mice in each group were killed for histopathological analysis. The remaining four mice in each group were used for functional analysis of the liver during the recovery period (2 weeks after the last liposome complex treatment).

### 2.10 Histopathological analysis

Tissue sections were stained with haematoxylin and eosin (H&E), Masson and Sirius red stains to examine the pathological changes in liver tissue as per the standard procedures.^22^ To assess the extent of chronic liver damage, an independent pathologist performed semiquantitative pathological grading (scores 0 to 18) and staging (scores 0 to 6), as described previously.^23^

### 2.11 Serum biochemistry

The serum was separated from the whole blood by centrifugation. The concentration of alanine aminotransferase (ALT), aspartate aminotransferase (AST), hyaluronate (HA) and total bilirubin (TBIL) were measured, as per the manufacturer's instruction (Nanjing Jiancheng).

### 2.12 Immunohistochemical staining

The paraffin sections of mouse liver were stained by standard immunohistochemical procedures, as reported previously.^18^ Briefly, after deparaffinization, hydration and antigen retrieval, slides were incubated with anti‐mouse α‐SMA (Alpha‐smooth muscle actin) primary polyclonal antibody (1:200, Abcam) overnight at 4°C. Subsequently, the slides were incubated with an HRP‐conjugated secondary antibody (1:2000, Abcam) for 1 h at room temperature. The immunohistochemical reaction was visualized by 3,3′‐diaminobenzidine‐tetrahydrochloride‐dihydrate (DAB) staining. The immunohistochemical staining images were captured by the digital TV camera system (AxioCam, Carl Zeiss, Inc). The percentage of stained areas in the digital photomicrographs were evaluated using an automated software analysis program (KS400, Carl Zeiss, Inc).

### 2.13 qRT‐PCR

Total RNA was extracted from mouse liver tissue using TRIzol reagent (Invitrogen). Later, the RNA was reverse transcribed to cDNA by SuperScript IV First‐Strand Synthesis Kit (Invitrogen). We measured the expression levels of the TLR4, collagen I, matrix metalloproteinase 2 (MMP‐2), MMP‐9, tissue inhibitor of metalloproteinase 1 (TIMP‐1), TIMP‐2, p22phox, gp91phox, p40phox, p47phox, p67phox and Rac‐1 by qRT‐PCR using gene‐specific primers and Green PCR Master Mix (Invitrogen). GAPDH was used as an internal control. The primers are listed in Table S1.

### 2.14 Western blot

Briefly, proteins were extracted from murine liver and primary HSCs using RIPA lysis buffer. 20 μg of protein samples were separated by 10% sodium dodecyl sulphate‐polyacrylamide gel electrophoresis (SDS‐PAGE). Subsequently, the separated proteins were transferred to PVDF membranes and probed with TLR4, Desmin, and albumin antibodies. The bands were visualized using Pierce™ ECL, Western blotting substrate and iBright™ CL1000 Imager (Thermo Fisher Scientific Inc). Quantitative densitometric analysis was conducted using the iBright™ analysis software. Experiments were repeated three times.

### 2.15 Nuclear factor‐kappa B (NF‐κB) activity assay

We investigated the transcriptional activity of NF‐κB in HSCs by dual‐luciferase reporter assay, as mentioned in the previous studies.^18^ Briefly, we cotransfected the TLR4‐shRNA and the NF‐κB luciferase reporter plasmids in HSCs via the VitA‐coupled liposomes. It was followed by the stimulation of cotransfected HSCs with 500 ng/mL lipopolysaccharide (LPS). Furthermore, 12 hours after this treatment, the cells were lysed, and intracellular luciferase activity was determined in the supernatant by the Dual‐Glo Luciferase Assay system as per the manufacture's instruction (Promega).

### 2.16 ELISA

We quantified the pro‐inflammatory cytokine levels in HSCs by ELISA kit (R&D), as per the manufacturer's instructions. Concisely, HSCs were seeded in 12‐well plates and incubated overnight. Later, they were transfected with the TLR4‐shRNA or control plasmid via the VitA‐coupled liposomes and incubated at 37°C and in humidified air with 5% CO_2_ for 96 hours. It was followed by the stimulation of the transfected HSCs with 500 ng/mL LPS for 48 hours. The cell culture supernatant was collected by centrifugation at 1500 RPM, and pro‐inflammatory cytokine levels were detected in it with an ELISA kit.

### 2.17 Measurement of reactive oxygen species (ROS)

Superoxide production in HSCs was detected by Cell Meter™ Fluorimetric Intracellular Total ROS Activity Assay Kit, according to the manufacture's instruction. Precisely, ROS Brite™ 670 working solution was added to the culture medium and incubated for 20 minutes at 37°C. Later, the HSCs were washed with PBS, and fluorescence was detected by fluorescence microscopy. In situ production of mitochondrial ROS from the mice liver cells was measured by Amplex Red Hydrogen Peroxide/Peroxidase Assay Kit (Thermo Fisher), as mentioned in the previous investigation.^24^ In brief, mitochondria were isolated by conventional differential centrifugation from the liver homogenates. The ROS production from mitochondrial complex I, complex III and reverse flow of electrons was detected by the fluorescence microplate reader.

### 2.18 NADPH oxidase assay

We extracted the total protein from mouse liver tissue lysate and quantified it by Bio‐Rad protein assay reagent as per the previous study.^25^ The liver NADPH oxidase was detected by EnzyChrom NADP/NADPH assay kit (BioAssay Systems), as per the manufacturer's instructions.

### 2.19 Statistical analysis

All the statistical tests were performed on the SPSS version 15.0 software. We employed one‐way ANOVA followed by Tukey's test for the statistical comparisons, and *P*‐value <.05 was considered as statistically significant.

## 3 RESULTS

### 3.1 Augmentation of DNA transfection and interference efficiency by the VitA‐coupled liposomes in HSCs

**FIGURE 1:**
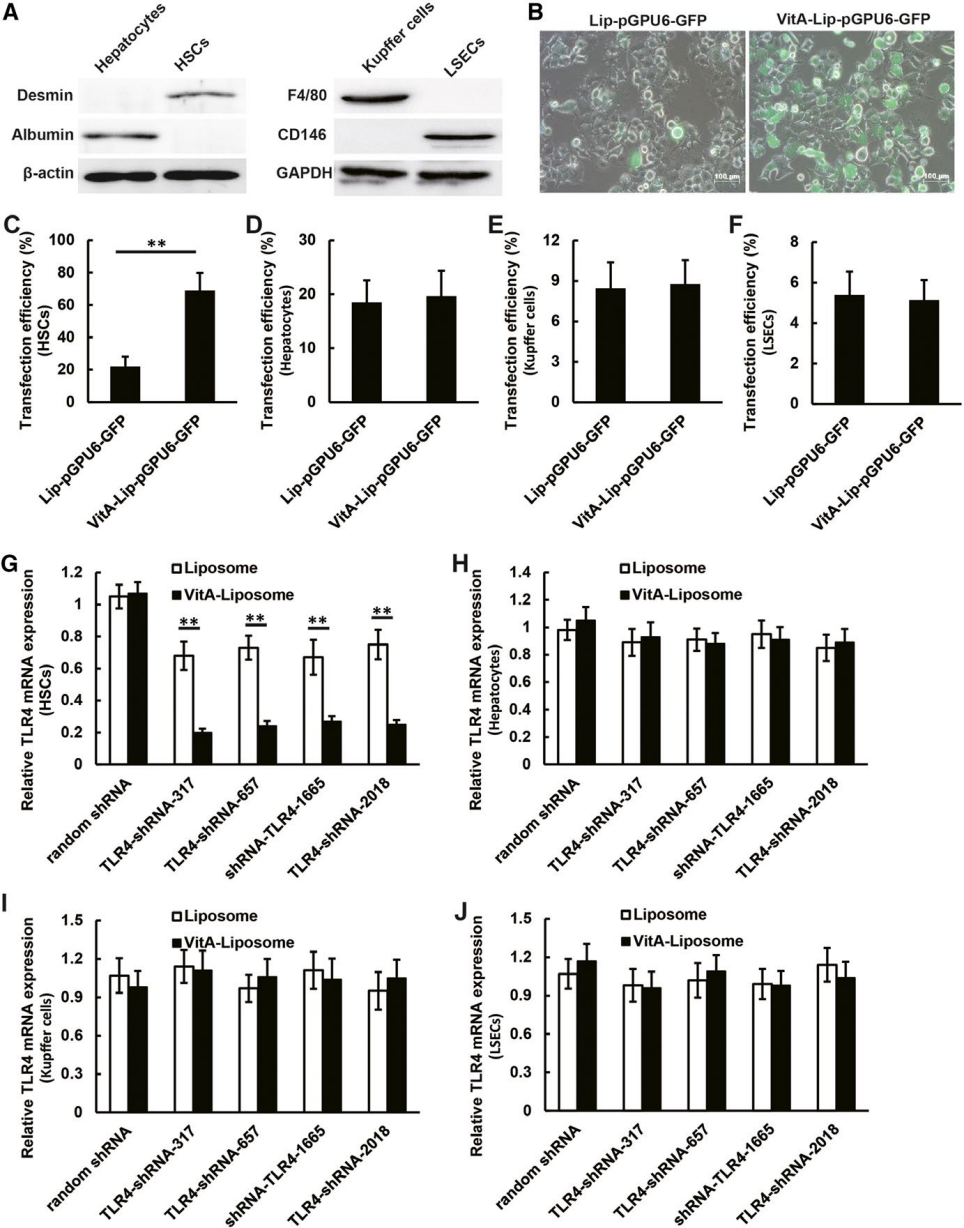
Transfection and interference efficiency of the VitA‐lip‐TLR4‐shRNA complex in the cultured primary HSCs and hepatocytes. A, The isolated primary HSCs cultured for 7 d were positive for Desmin HSC marker and negative for albumin hepatocyte marker. The isolated Kupffer cells and LSECs were found to be positive for F4/80 and CD146, respectively. B, Pictorial representation of the transfection efficiency of the lip‐pGPU6/GFP and the VitA‐lip‐pGPU6/GFP in primary murine HSCs. C, The transfection efficiency of the VitA‐lip‐pGPU6/GFP complex was higher as compared to the lip‐pGPU6/GFP complex in HSCs in vitro (*P* < .01). The transfection efficiency of two kinds of liposomes in primary murine (D) hepatocytes, (E) Kupffer cells and (F) LSECs was similar, which was lower than 20%, 9% and 6%, respectively. G, The interference efficiency of four candidate TLR4‐shRNA plasmids encapsulated within the VitA‐coupled liposomes in the HSCs was examined using qRT‐PCR. TLR4‐shRNA plasmids decreased the TLR4 expression in murine HSCs (**P < .01). Four VitA‐lip‐TLR4‐shRNA complex candidates did not suppress the TLR4 mRNA expression in primary murine (H) hepatocytes, (I) Kupffer cells and (J) LSECs

We detected the specific cell markers in the isolated HSCs. The isolated primary HSCs cultured for 7 days were found to be positive for Desmin HSC marker and negative for albumin hepatocyte marker. The isolated Kupffer cells and LSECs were found to be positive for F4/80 and CD146, respectively (Figure 1A). We found that the transfection efficiency of the VitA‐lip‐pGPU6/GFP complex was higher than that of the lip‐pGPU6/GFP complex in HSCs (*P* < .01) as depicted in Figure 1B,C. It suggests that the transfection efficiency can be increased by 2.5‐fold with the help of the VitA‐coupled liposome‐based transfection reagents. However, the transfection efficiency of VitA‐coupled liposomes and uncoupled liposomes in murine primary hepatocytes, Kupffer cells and LSECs were similar, which were lower than 20%, 9% and 6%, respectively (Figure 1D‐F). Next, we have screened RNA interference efficiency via four TLR4‐shRNA complex candidates. We found that the plasmids expressing four candidates TLR4‐shRNA‐encapsulated within VitA‐coupled liposomes could decrease the expression of the TLR4 in murine HSCs (*P* < .01) (Figure 1G). However, four VitA‐lip‐TLR4‐shRNA complex candidates did not decrease the TLR4 expression in primary murine hepatocytes, Kupffer cells and LSECs (Figure 1H‐J). In this study, we used pooled shTLR4‐317 and shTLR4‐657 at a molar ratio of 1:1 to treat liver fibrosis.

### 3.2 Pharmacokinetics and organic distribution of the VitA‐lip‐TLR4‐shRNA

**FIGURE 2:**
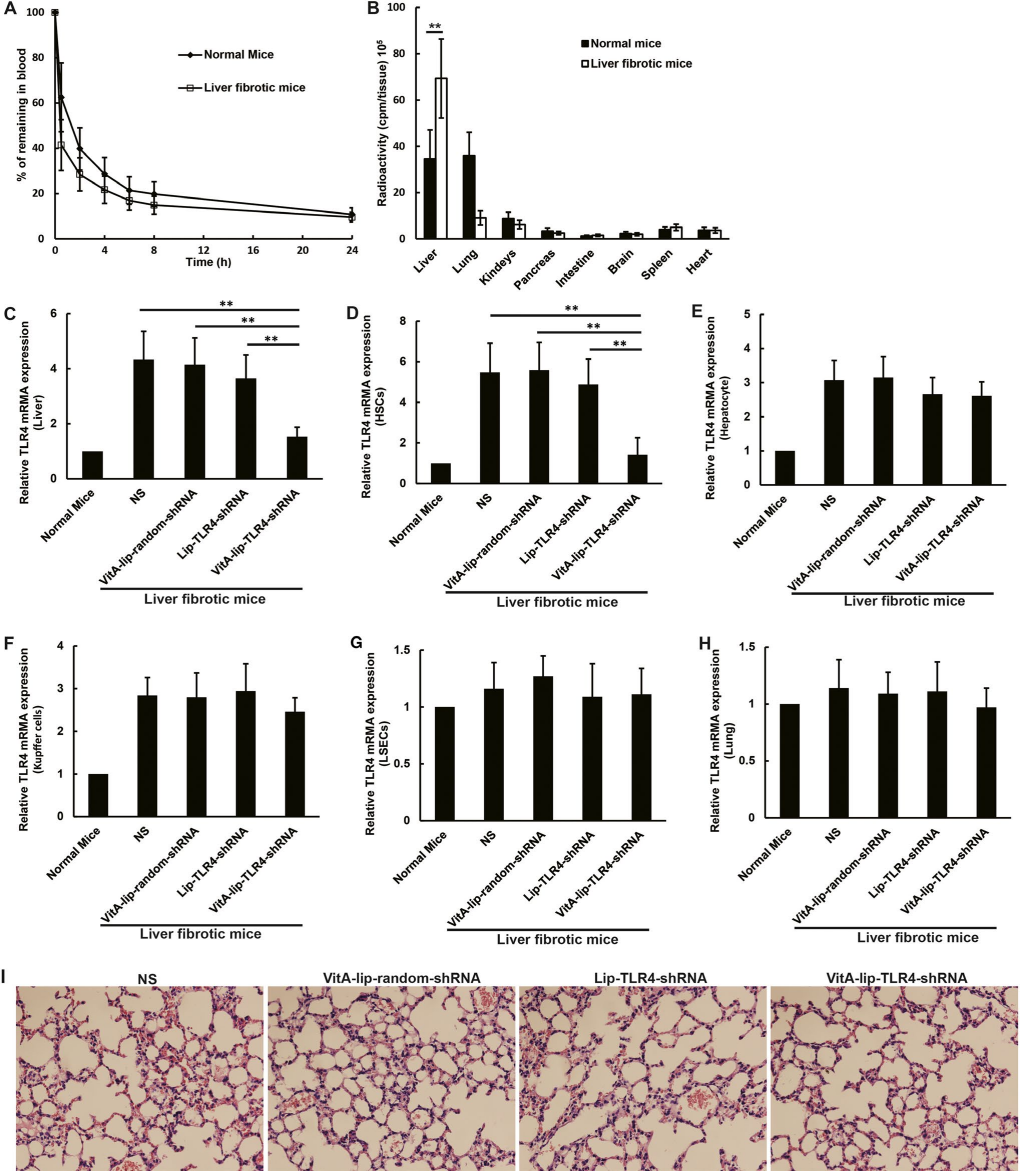
Pharmacokinetics, organ distribution and interference efficiency of the VitA‐lip‐TLR4‐shRNA complex in mice (n = 4 per group). A, The half‐life of the [^3^H]VitA‐lip‐TLR4‐shRNA complex in blood was around 23 and 72 min for liver fibrosis afflicted and normal mice, respectively. B, The [^3^H]VitA‐lip‐TLR4‐shRNA complex was primarily distributed in the liver and lung in normal mice. The [^3^H]VitA‐lip‐TLR4‐shRNA complex was distributed only in the CCl_4_‐induced fibrotic liver of mice. C, In the CCl_4_‐induced fibrotic liver of mice, the VitA‐lip‐TLR4‐shRNA treatment decreased the TLR4 mRNA expression as compared to the control group (*P* < .01). The mRNA levels were normalized by the expression of GAPDH and were expressed as fold change relative to the normal mice. D, The TLR4 mRNA level in primary murine HSCs isolated from the liver of VitA‐lip‐TLR4‐shRNA‐treated mice was significantly lower than the control mice (*P* < .01). The TLR4 mRNA level in primary murine (E) hepatocytes, (F) Kupffer cells and (G) LSECs isolated from VitA‐lip‐TLR4‐shRNA‐treated mice was similar to the control groups. H, No significant difference was seen in the TLR4 mRNA expression in the mice lung between these groups. **P < .01. I, Histopathological changes in lungs post‐VitA‐lip‐TLR4‐shRNA treatment. Representative images of H&E staining for lung (×200)

We examined the pharmacodynamics and organic distribution of the VitA‐lip‐TLR4‐shRNA complex and [^3^H]VitA‐lip‐TLR4‐shRNA by injecting them into the mice with normal or CCl4‐induced fibrotic liver. The half‐life of the [^3^H]VitA‐lip‐TLR4‐shRNA in blood was around 23 and 72 min for fibrotic liver mice and normal mice, respectively (Figure 2A). The primary site of the [^3^H]VitA‐lip‐TLR4‐shRNA complex distribution was the liver and lung in normal mice, 24 hours post‐injection. However, the [^3^H]VitA‐lip‐TLR4‐shRNA complex was distinctively distributed in the liver of mice with CCl4‐induced fibrotic liver. The radioactivity in the liver of the mice with the CCl4‐induced fibrotic liver was more as compared to the control mice (*P* < .01) (Figure 2B).

### 3.3 The TLR4 gene silencing efficiency of the VitA‐lip‐TLR4‐shRNA in mice

We observed a significant augmentation of the TLR4 expression in mice with the CCl4‐induced fibrotic liver compared with normal mice. Subsequently, we evaluated the gene silencing efficiency of the VitA‐lip‐TLR4‐shRNA complex in mice. The TLR4 mRNA level in organs was detected with the help of qRT‐PCR. We observed that in mice with CCl4‐induced fibrotic liver, the TLR4 mRNA level in the liver of VitA‐lip‐TLR4‐shRNA‐treated mice was markedly lower as against the control mice (*P* < .01) (Figure 2C). Furthermore, we detected TLR4 expression in isolated HSCs, hepatocytes, Kupffer cells and LSECs, post‐plasmid‐liposome complex treatment in vivo. The TLR4 mRNA level in primary murine HSCs isolated from the liver of VitA‐lip‐TLR4‐shRNA‐treated mice was significantly lower than the control (*P* < .01) (Figure 2D). However, the TLR4 mRNA level in primary murine hepatocytes, Kupffer cells and LSECs isolated from the VitA‐lip‐TLR4‐shRNA‐treated mice was similar to the control (Figure 2E‐G). Besides, no significant differences were found in the TLR4 mRNA expression in the lung between the groups of mice (Figure 2H). Moreover, alveolar cell apoptosis or necrosis and inflammatory cell infiltration were not observed in the lung tissues post‐VitA‐lip‐TLR4‐shRNA treatment (Figure 2I).

### 3.4 Lower progression of liver fibrosis and restoration of liver function following the VitA‐lip‐TLR4‐shRNA treatment

**FIGURE 3:**
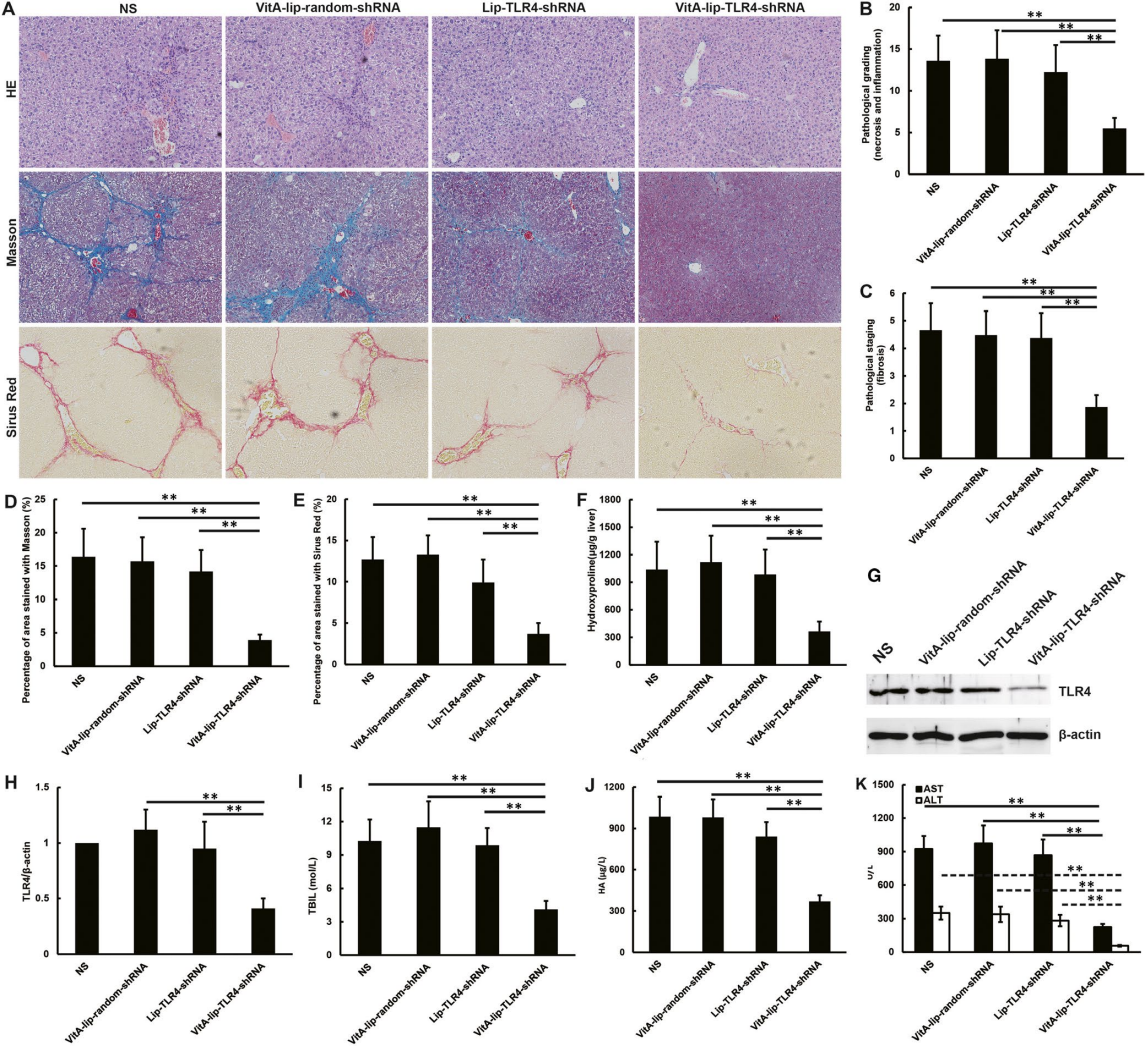
The slower progression of liver fibrosis and liver function restoration after VitA‐lip‐TLR4‐shRNA complex treatment. A, Images representing H&E, Masson, and Sirius red staining of the mice liver. The pathological (B) grading (necrosis and inflammation) and (C) staging (fibrosis and architectural alteration) were suppressed in VitA‐lip‐TLR4‐shRNA‐treated mice. D, Masson positive staining area and; E, Sirius red positive staining area assessed by computerized image analysis. Data were obtained from six randomly selected fields in each group. F, The VitA‐lip‐TLR4‐shRNA complex treatment decreased the hydroxyproline levels in the liver of the CCl_4_‐induced fibrotic liver of mice as compared to the control treatment groups. G, The TLR4 protein expression in the liver was analysed by Western blotting after administering 10 doses of VitA‐lip‐TLR4‐shRNA. Each pool included three samples. H, Quantitative densitometric analysis of Western blots. The VitA‐lip‐TLR4‐shRNA complex treatment significantly attenuated the increased serum level of serum (I) TBIL; (J) HA; (K) ALT and AST as compared to the control groups. **P < .01

We investigated the histopathological changes in the liver with the help of H&E, Sirius red and Masson staining procedure. H&E staining of liver paraffin sections demonstrated severe CCl4‐induced hepatic damage. VitA‐lip‐TLR4‐shRNA noticeably reduced hepatocyte damage and inflammatory cell infiltration. The pathological grading (necrosis and inflammation) and staging (fibrosis and architectural alteration) were both suppressed in the VitA‐lip‐TLR4‐shRNA‐treated mice (Figure 3A‐C). Furthermore, the Sirius red and Masson staining also validated that the VitA‐lip‐TLR4‐shRNA complex curtails the collagen deposition in the liver of CCL4‐induced fibrotic liver mice as compared to the control group (*P* < .01) (Figure 3A,D‐E). Similarly, the VitA‐lip‐TLR4‐shRNA complex treatment also alleviated the hydroxyproline levels in the liver of mice with CCL4‐induced fibrotic liver as compared to the liver of control mice (*P* < .01) (Figure 3F).

Toll‐like receptor 4 protein expression in the mouse liver was examined using Western blot analysis after administration of 10 doses of VitA‐lip‐TLR4‐shRNA. The outcome of this analysis showed that the TLR4 protein expression in the liver of VitA‐lip‐TLR4‐shRNA‐treated mice was significantly lower than control, and TLR4 expression was negatively correlated to the liver fibrosis progression (Figure 3G‐H).

The serum concentration of the ALT, AST, HA and TBIL were evaluated to investigate the restoration of liver function 2 weeks post‐last VitA‐lip‐TLR4‐shRNA complex treatment. The VitA‐lip‐TLR4‐shRNA treatment attenuated the serum levels of the ALT, AST, HA and TBIL as compared to the control groups (*P* < .01) (Figure 3I‐K).

### 3.5 The VitA‐lip‐TLR4‐shRNA inhibits HSCs activation and restores the balance between synthesis and degradation of ECM in mice afflicted with liver fibrosis

**FIGURE 4:**
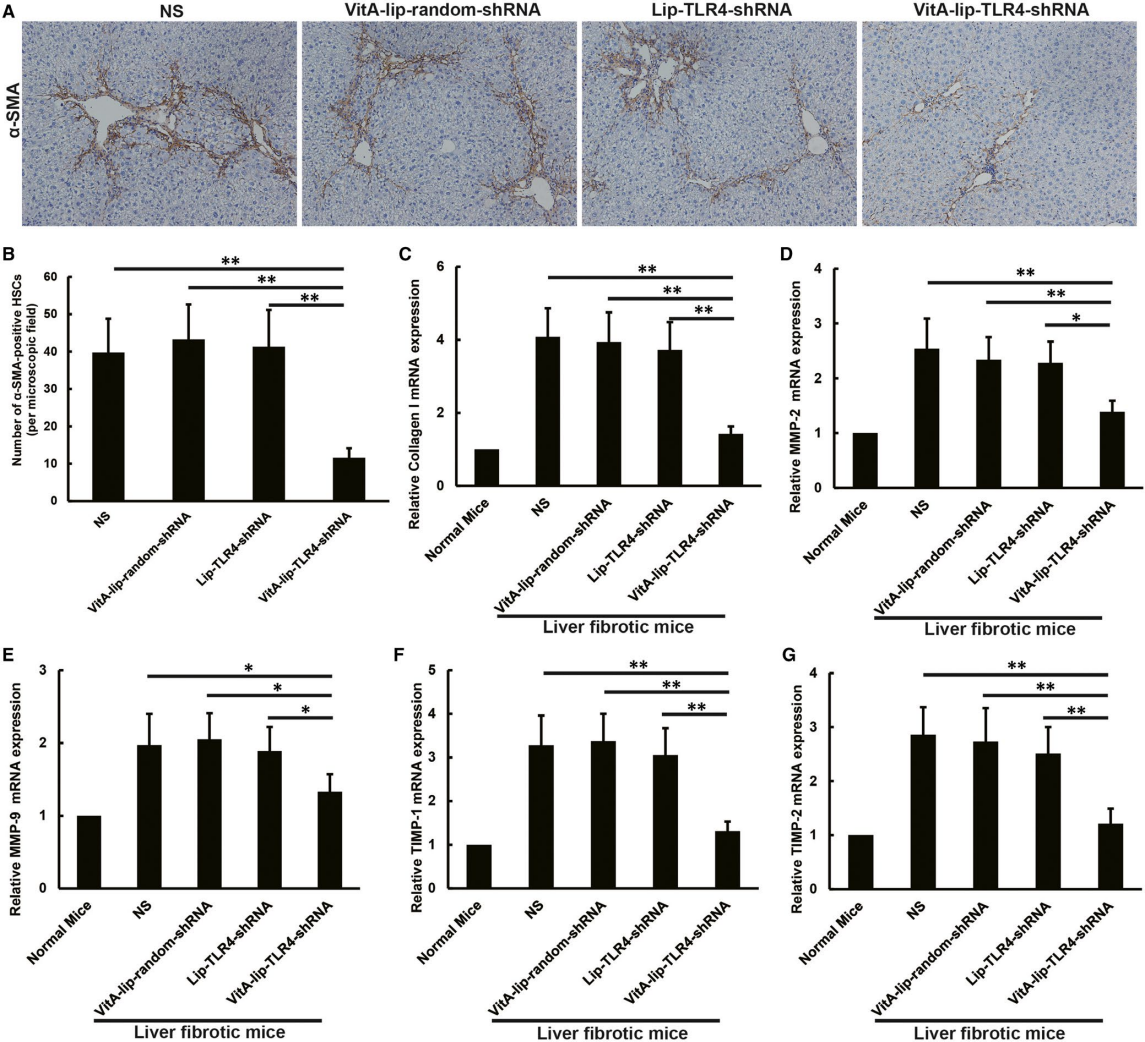
The VitA‐lip‐TLR4‐shRNA inhibited the HSCs activation and restored the balance between the ECM synthesis and degradation in the CCl_4_‐induced fibrotic liver in mice. A, Immunohistochemical staining of hepatic aHSCs. B, Statistical analysis of α‐SMA‐positive HSCs. The VitA‐lip‐TLR4‐shRNA complex remarkably decreased the number of α‐SMA‐positive HSCs in the liver as compared to the control treatment groups (*P* < .01). C, The VitA‐lip‐TLR4‐shRNA complex decreased the type I collagen mRNA expression in the CCl_4_‐induced fibrotic liver as against the control treatment groups (*P* < .01). The mRNA level of (D) MMP‐2; (E) MMP‐9; (F) TIMP‐1; and (G) TIMP‐2 was decreased in the liver of the CCl_4_‐induced fibrotic liver in mice after the VitA‐lip‐TLR4‐shRNA complex treatment as compared to the control treatment groups. The down‐regulation of TIMP‐1 and TIMP‐2 expression was more evident than that of MMP‐2 and MMP‐9 expression. The mRNA levels were normalized by the expression of GAPDH in each experiment and were expressed as fold change relative to the normal mice. **P < .01, *P < .05

Hepatic stellate cells activation is a crucial process in the onset of liver fibrosis. Conventionally, α‐SMA (Alpha‐smooth muscle actin) is treated as a specific molecular marker to detect activated HSCs (aHSCs). We checked its expression in liver HSCs by immunohistochemistry. The VitA‐lip‐TLR4‐shRNA complex treatment reduced the number of α‐SMA‐positive HSCs in fibrotic liver HSCs as compared to the liver of control mice (*P* < .01) (Figure 4A‐B). aHSCs have the potential to transform into myofibroblast‐like cells, which majorly act as ECM‐producing cells and promote fibrotic liver progression. Furthermore, we examined the effects of the TLR4 gene silencing in aHSCs induced ECM synthesis and degradation. It was assessed by investigating the mRNA levels of the type I collagen, MMP‐2, MMP‐9, TIMP‐1 and TIMP‐2 in the fibrotic liver cellular extract with the help of qRT‐PCR. We found that the VitA‐lip‐TLR4‐shRNA complex treatment noticeably reduced the mRNA levels of type I collagen in the liver (*P* < .01) (Figure 4C). Besides, we also observed escalated mRNA levels of MMP‐2, MMP‐9, TIMP‐1 and TIMP‐2 in the CCl4‐induced fibrotic liver cells of mice. However, the VitA‐lip‐TLR4‐shRNA complex treatment reduced the MMP‐2, MMP‐9, MMPs inhibitor, TIMP‐1 and TIMP‐2 mRNA level in the liver of mice with CCl4‐induced fibrotic liver. However, the TIMP‐1 and TIMP‐2 expression decreased more than that of the MMP‐2 and MMP‐9 expression.

### 3.6 TLR4 silencing inhibits NF‐κB transcriptional activity and suppresses the release of pro‐inflammatory cytokines in LPS treated HSCs exposed, in vitro

**FIGURE 5:**
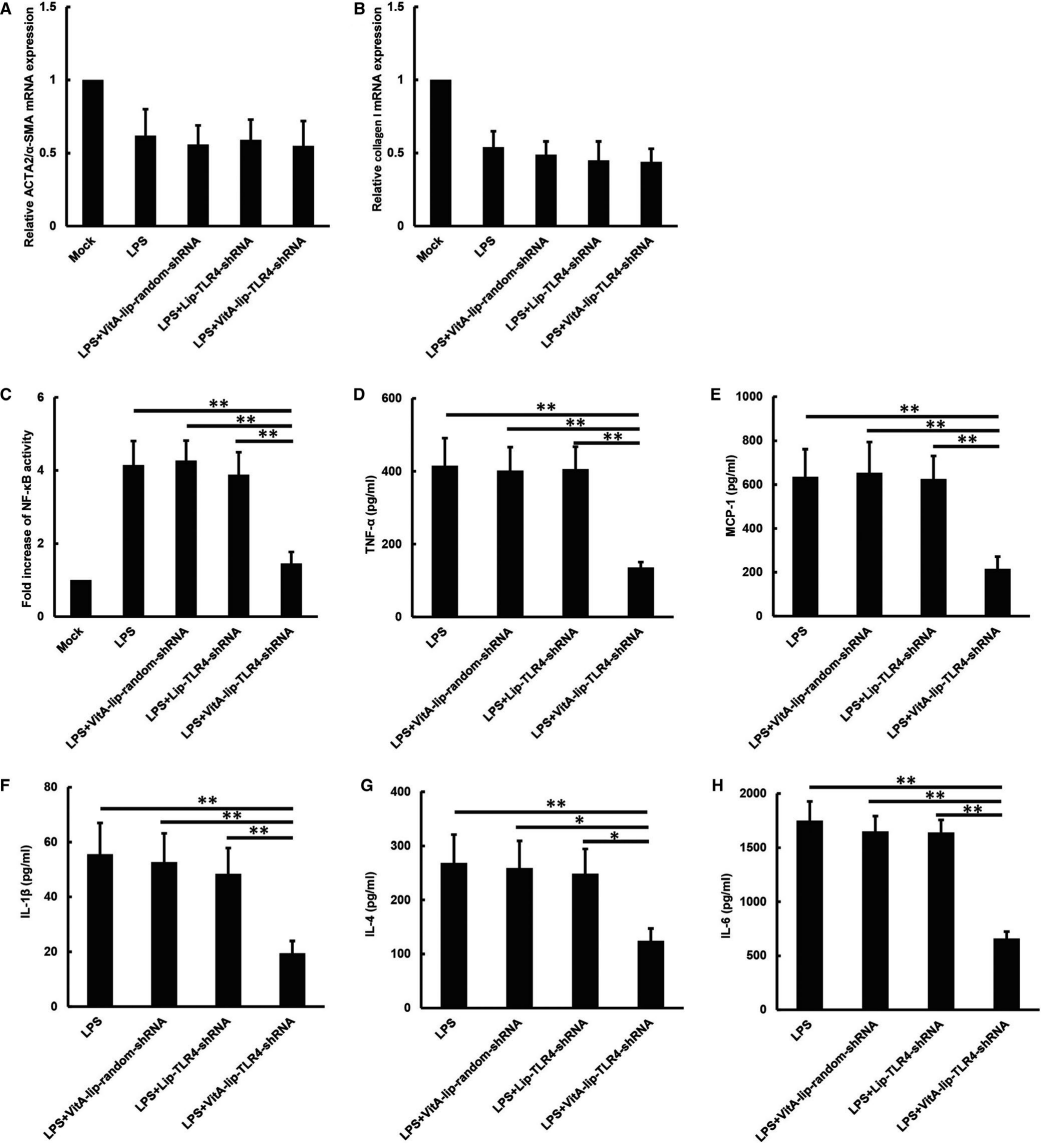
The TLR4 gene silencing inhibits NF‐κB transcriptional activity and attenuates pro‐inflammatory cytokines secretion in LPS treated HSCs in the in vitro condition. A, LPS down‐regulated the ACTA2/α‐SMA expression in activated HSCs. The TLR4 gene silencing did not significantly reduce the expression of ACTA2/α‐SMA in the LPS activated HSCs. B, LPS down‐regulated the collagen I expression in activated HSCs. The TLR4 gene silencing did not significantly reduce the expression of collagen I in the LPS activated HSCs. C, The TLR4 silencing inhibited NF‐κB activity in LPS‐stimulated HSCs as against the control groups; (D) TNF‐α; (E) MCP‐1; (F) IL‐1β; (G) IL‐4; (H) IL‐6 secretion from LPS‐stimulated HSCs after treatment with VitA‐lip‐TLR4‐shRNA complex was significantly reduced as compared to the control treatment groups. **P < .01, *P < .05

The TLR4 stimulation leads to the activation of the NF‐κB signalling pathway, which in turn induces the over‐expression of inflammatory cytokine genes. Firstly, we detected the α‐SMA and collagen I expression in LPS treated HSCs in vitro. Interestingly, LPS down‐regulated the ACTA2/α‐SMA and collagen I expression in activated HSCs. Furthermore, the ACTA2/α‐SMA and collagen I mRNA expression did not reduce significantly because of the TLR4 gene silencing in the LPS activated HSCs (Figure 5A‐B). Subsequently, we detected the NF‐κB activity and inflammatory cytokine gene transcription in cultured HSCs. As demonstrated in Figure 5C, the TLR4 silencing significantly inhibited the NF‐κB activity in LPS treated HSCs as compared to the control (*P* < .01). The expression of NF‐κB‐regulated genes encoding inflammation‐associated molecules and cytokines such as TNF‐α, MCP‐1, IL‐1β, IL‐4 and IL‐6 was evaluated by ELISA. We observed that the TLR4 gene silencing significantly reduced the levels of these cytokines in the LPS activated HSCs (Figure 5D‐H).

### 3.7 VitA‐lip‐TLR4‐shRNA attenuates ROS production in vitro and in vivo

**FIGURE 6:**
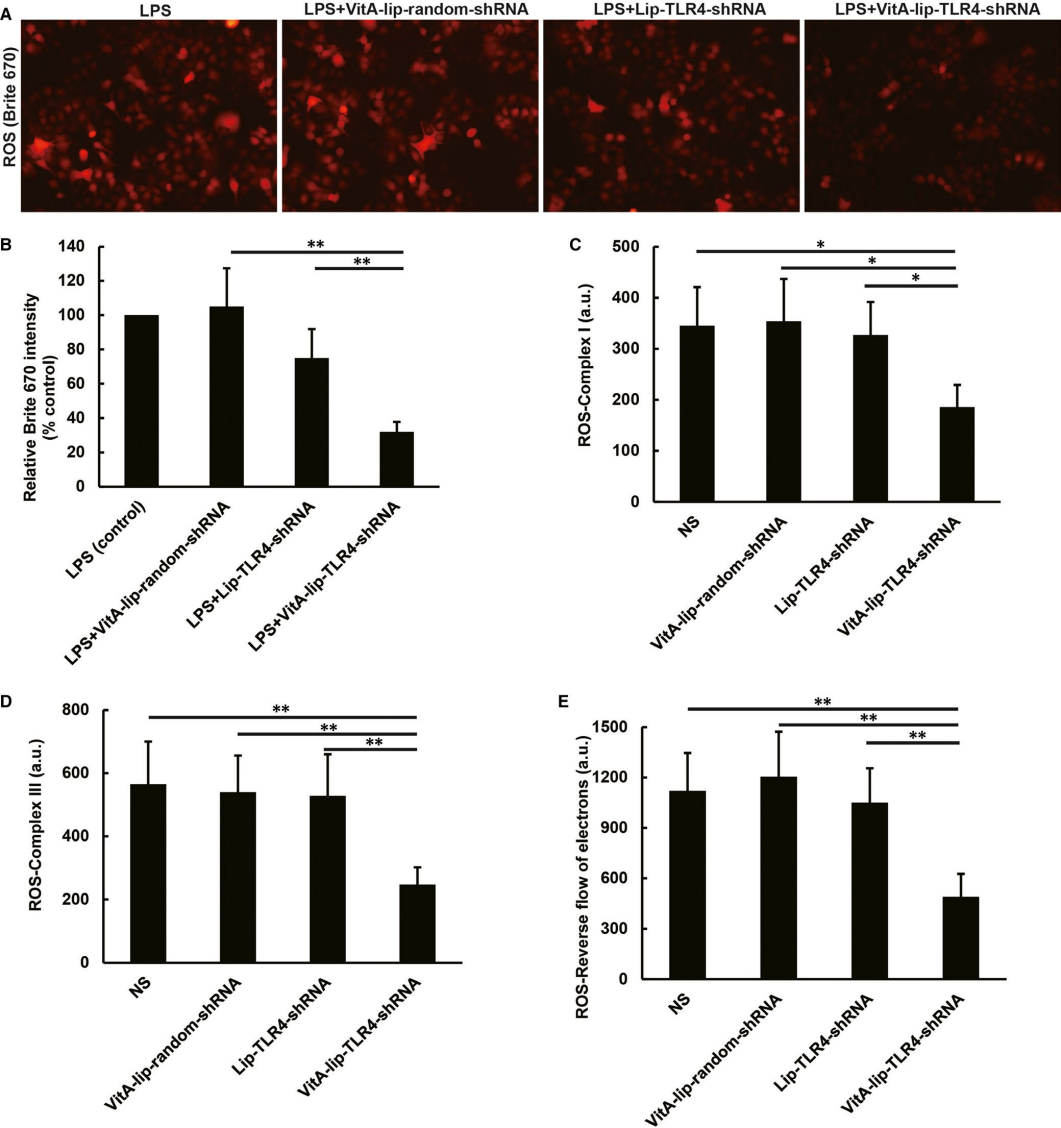
The VitA‐lip‐TLR4‐shRNA complex attenuates the mitochondrial ROS production in the in vitro and in vivo condition. A, Fluorescent microscopic images of mitochondrial‐derived ROS. B, Statistical analysis of ROS production. The TLR4 silencing attenuated the LPS‐induced ROS production in cultured HSCs. The VitA‐lip‐TLR4‐shRNA complex treatment decreased the mitochondrial ROS production derived from (C) mitochondrial complex I, (D) complex III, (E) in reverse flow of electrons in the CCl4‐induced hepatic fibrosis in mice as compared to the control group of mice. **P < .01, *P < .05

**FIGURE 7:**
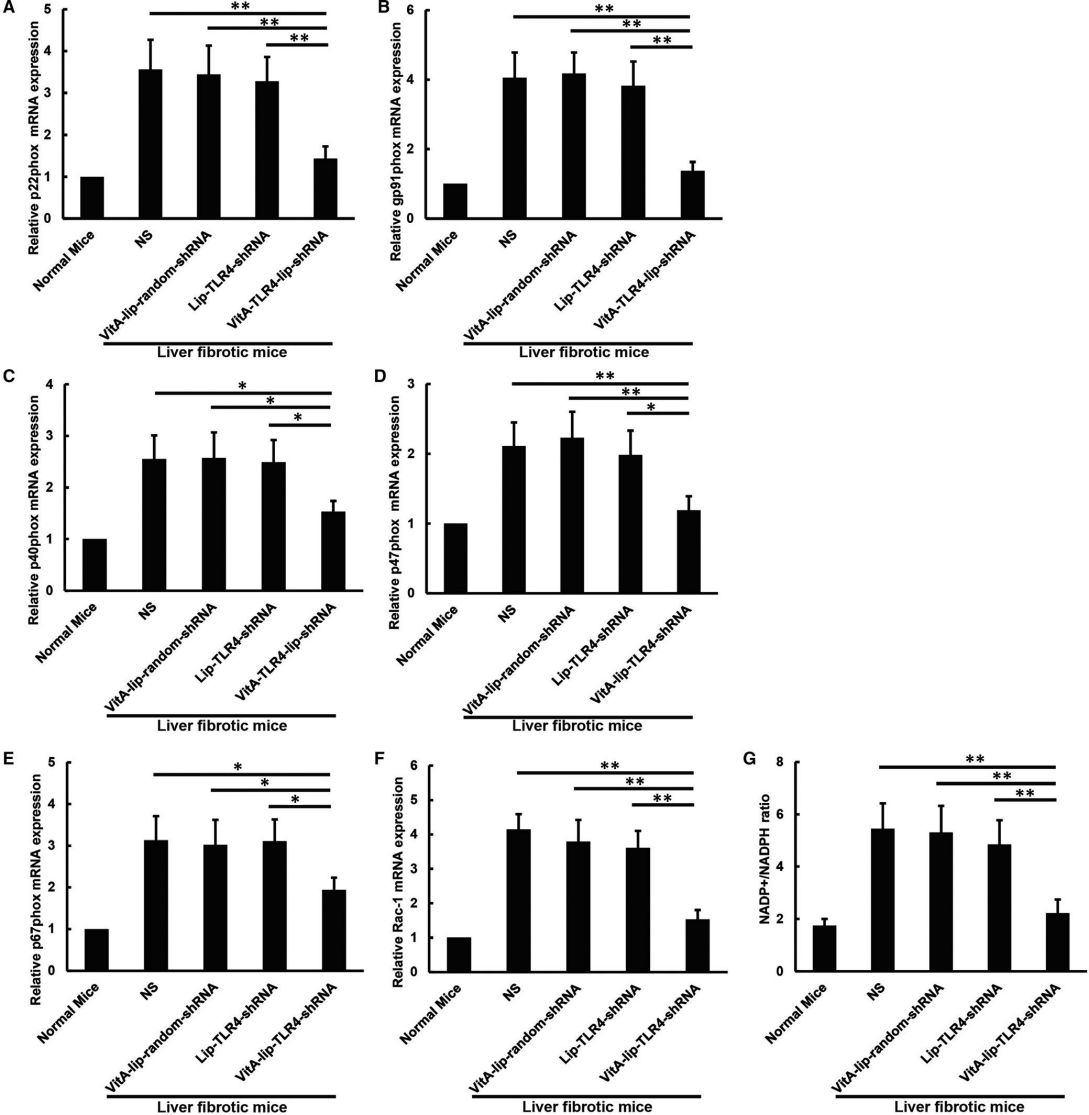
The VitA‐lip‐TLR4‐shRNA complex attenuated the NADPH oxidase system. A, NADPH oxidase subunits p22phox; (B) gp91phox; (C) p40phox; (D) p47phox; (E) p67phox; (F) Rac‐1 mRNA expression in the CCl4‐induced hepatic fibrosis mice as compared to the control treatment groups. The mRNA levels were normalized by the expression of GAPDH in each experiment and were expressed as fold change relative to the normal mice. G, The VitA‐lip‐TLR4‐shRNA complex treatment decreased the NADP/NADPH conversion rate in CCl4‐induced hepatic fibrosis mice as compared to the control group of mice. **P < .01, *P < .05

LPS treated HSCs demonstrated excessive ROS production. However, the TLR4 gene silencing attenuated this escalated ROS production in LPS‐stimulated HSCs (*P* < .01) (Figure 6A‐B). The VitA‐lip‐TLR4‐shRNA complex treatment notably reduced the mitochondrial ROS production from mitochondrial complex I, complex III, and in reverse flow of electrons in the CCl4‐induced fibrotic liver of mice as compared to the liver of control mice (Figure 6C‐E). The non‐mitochondrial ROS is primarily produced in the NADPH oxidases inside the cells. The NADPH oxidase complex consists of two transmembrane proteins (p22phox and gp91phox) and four cytoplasmic proteins (p40phox, p47phox, p67phox and Rac‐1). We found that the VitA‐lip‐TLR4‐shRNA complex treatment diminished the total mRNA expression of the NADPH oxidase subunits in the CCl4‐induced fibrotic liver of mice as compared to the liver of control mice (Figure 7A‐F). Consistent with the decreased mRNA levels of the NADPH oxidase complex, the VitA‐lip‐TLR4‐shRNA complex treatment reduced the NADP/NADPH synthesis rate in the CCl4‐induced fibrotic liver of mice as compared to the liver of control mice (*P* < .01) (Figure 7G).

## 4 DISCUSSION

Hepatic fibrosis is a severe pathological condition, which gradually develops into liver cirrhosis or hepatocellular carcinoma.^26^ A massive activation and proliferation of quiescent HSCs were reported in the hepatic fibrogenesis. HSCs are primarily activated by autocrine and paracrine factors, such as cytokines, growth factors, lipid peroxides and ROS.^5^ The activated HSCs play a crucial role in liver fibrosis onset and progression via cytokine secretion and fibrotic ECM formation after encountering the liver injury.^27^

Recent reports suggest that the gut‐liver axis may promote liver fibrosis.^28^ A highly permeable intestinal mucosal barrier absorbs a significant amount of bacterial LPS. However, hepatocyte injury hampers the liver's function of bacterial LPS detoxification.^10^ Exogenous intestinal bacterial components, such as LPS and endogenous substances derived from dead or damaged cells, induce the TLR4 activation.^27^, ^28^

A cumulative action of LPS and these endogenous substances results in the TLR4 mediated HSCs activation, which finally culminates in liver fibrosis.^27^, ^29^ Thus, prevention and reversal of TLR4 mediated HSCs activation might serve as an effective therapeutic strategy in the management of liver fibrosis. Previous studies have demonstrated that TAK‐242, a small molecule and a TLR4 inhibitor, suppressed liver injury and reduced organ fibrosis in animal models.^30^, ^31^ TLR4 is expressed on the surface of HSCs and immunocytes, such as neutrophils, monocytes, dendritic cells and macrophages. To avoid the non‐specific inhibition of small molecule TLR4 inhibitors on the immunocytes activity, in the current study, we mitigated the HSCs activation by HSCs specific liposomes with the TLR4 shRNA to treat the CCl4‐induced fibrotic liver in mice.

The major challenge in RNAi therapeutics is its delivery system. The RNAi delivery system can be categorized into a viral and non‐viral delivery system.^32^ The viral delivery system primarily comprises adenoviruses, adeno‐associated virus and lentivirus. Besides being efficient in cell transduction, these viral vectors have an impeccable ability to enter the nucleus. Thus, the viral vectors efficiently express shRNA or miRNA and regulate the target gene expression.^33^ However, the major limitation encountered in the shRNA or miRNA approach is toxicity and altered host immune response, as well as insertional mutagenesis.^34^ The non‐viral delivery systems are safer as compared to the viral delivery systems. The current non‐viral delivery systems entail lipid, polymer and peptide‐based carriers.^32^ In the current research study, cationic liposomes were investigated for the introduction of the TLR4 shRNA inside the aHSCs.

Hepatic stellate cells play a major role in VitA intake through RARs present on the HSCs surface.^35^ Sato et al^15^ demonstrated that VitA‐coupled liposomes are specifically taken up by aHSCs in the fibrotic liver. The VitA‐coupled liposomes have to be applied to deliver siRNAs for the treatment of liver fibrosis.^15^, ^36^, ^37^ In this study, we attempted to deliver shRNA to aHSCs using VitA‐coupled cationic liposomes. The outcome of this study demonstrated that the [^3^H]VitA‐lip‐TLR4‐shRNA was primarily taken up by the liver in the CCl4‐induced fibrotic liver of mice. It led to a significantly shorter half‐live of the [^3^H]VitA‐lip‐TLR4‐shRNA in the blood of the fibrotic liver of mice in contrast to normal mice. We also found that the VitA‐coupled liposomes delivered the TLR4 shRNA to aHSCs more efficiently than the uncoupled liposomes. Besides, it was also evident that the VitA‐lip‐TLR4‐shRNA complex was primarily distributed in the liver and lung of normal mice. The high plasmid‐liposome complex was distributed in the lung of normal mice, which can be attributed to the rich network of blood vessels and high macrophage count in the lung.^38^ When the plasmid‐liposome complex was administered intravenously, it was phagocytosed and degraded by macrophages after reaching the pulmonary microvessels. Even after a high plasmid‐liposome complex distribution in the lung, signs of alveolar cell injury and inflammatory cell infiltration in the lung were not evident post‐VA‐lip‐shRNA‐TLR4 treatment. Moreover, no significant difference was observed in the TLR4 mRNA expression in the lungs of different mice groups. Interestingly, the distribution of [^3^H]VitA‐lip‐TLR4‐shRNA in the lung of fibrotic liver mice was significantly lower than that of normal mice. HSCs activation and proliferation in fibrotic liver mice led to [^3^H]VitA‐lip‐TLR4‐shRNA uptake by a large number of activated HSCs. However, the total concentration of injected [^3^H]VitA‐lip‐TLR4‐shRNA remained unchanged. The [^3^H]VitA‐lip‐TLR4‐shRNA distribution in the fibrotic liver was significantly more than that in the normal liver. Also, the VitA‐lip‐TLR4‐shRNA distribution in the lungs of the mice with liver fibrosis was significantly less than the lungs of normal mice.

Toll‐like receptor 4 expressed by hepatocytes is crucial for inflammation and fibrosis of the liver.^39^ TAK‐242, TLR4 inhibitor ameliorates liver injury and systemic inflammation in rodent models of liver failure.^40^ Furthermore, we investigated the VitA‐lip‐TLR4‐shRNA uptake by hepatocytes and the anti‐fibrotic effect of TLR4 silencing in hepatocytes. In the current study, although the TLR4 expression was up‐regulated in both primary murine HSCs and hepatocytes isolated from the fibrotic liver, reduced expression of TLR4 was found only in primary HSCs isolated from the fibrotic liver of VitA‐lip‐TLR4‐shRNA‐treated mice. It might be because of the low transfection efficiency of VitA‐coupled liposomes in hepatocytes.

Activated HSCs and extracellular matrix deposition is the pathological characteristic of liver fibrosis.^41^ The TLR4s are chiefly expressed on the surface of HSCs in the liver. The activation of the TLR4 signalling pathway plays a crucial role in liver fibrosis.^42^ As shown in the previous study, the TLR4 down‐regulation in the TLR4 knockout mice alleviated liver fibrosis in the experimental liver injury model.^5^ Besides, the administration of andrographolide inhibited the TLR4 expression and ameliorated the CCl4‐induced liver fibrosis in mice.^41^ Also, mogroside IVE mediated down‐regulation of the TLR4 signalling pathway inhibited the activation of HSCs and attenuated liver fibrosis in mice.^43^ In the present study, we have shown that the VA‐lip‐TLR4‐shRNA administration significantly inhibited the HSCs activation and slowed down the ECM deposition in the liver by TLR4 silencing and thus recovered the liver function.

The two crucial factors that regulate ECM synthesis and their degradation are MMPs and TIMPs. Previous studies have shown that the imbalanced activities of these factors contribute significantly to the pathology of liver fibrosis.^44^ We found that although TLR4 silencing diminished the expressions of MMPs and TIMPs, a higher fold reduction of TIMPs was witnessed than the MMPs. The outcome of this study suggests that the TLR4 silencing accelerated ECM degradation and reduced ECM synthesis, and ECM synthesis was at a slower rate than ECM degradation.

The impact of circulating LPS on fibrogenesis in the CCl4 model of this study remains controversial. The previous study reported an elevated concentration of serum LPS in CCl4‐treated TLR4 wild and mutant mouse models of fibrogenesis. Furthermore, the degree of liver fibrosis in TLR4 mutant mice was lower than that in TLR4 wild‐type mice after eight doses of CCl4 administration.^45^ The bacterial microflora (LPS) of the intestine and TLR4 promotes hepatic fibrogenesis. However, the serum LPS concentration of CCl4‐treated TLR4‐wild type, TLR4‐knockout and normal mice was identical.^46^ The role of TLR4 in CCl4‐induced fibrosis was independent of LPS. The contradictory results might be because of the different experimental conditions. In addition, mice injected with CCl4 in advanced stages of liver fibrosis demonstrated augmented LPS levels because of the bacterial overgrowth.^47^ Thus, the disparity in serum's LPS concentration might be because of different blood‐sample collection time‐points in these studies. As mentioned previously, the liver's ability to detoxify bacterial LPS is hampered in advanced stages of liver fibrosis; however, the exogenous intestinal bacterial LPS induces the TLR4 activation.^28^ Interestingly, in this study, we observed LPS‐induced down‐regulation of ACTA2/α‐SMA and collagen I expression in activated HSCs. The outcome of our analyses was in line with the previous study.^46^ Moreover, TLR4 gene silencing in the LPS activated HSCs did not mitigate the ACTA2/α‐SMA and collagen I mRNA expression. However, TLR4 silencing inhibited NF‐κB transcriptional activity and suppressed the pro‐inflammatory cytokine's secretion in LPS treated HSCs in vitro. In the CCl4 model of the current study, the serum LPS concentration and its impact liver fibrogenesis demand further investigation.

The mitochondrial respiratory chain or NADPH oxidases are the primary sites of intracellular ROS synthesis. It serves as second messengers and plays a crucial role in cellular physiological and pathophysiological activities.^48^ Numerous studies have reported that excessive ROS production leads to aberrant liver tissue repair and liver fibrosis.^49^ An excessive ROS generation induces abnormal HSCs activation during liver damage. As shown in the previous studies, mitoquinone, a mitochondria‐targeted antioxidant, mitigated the ROS level, which in turn attenuated liver inflammation and fibrosis in cirrhotic rats.^50^ Inhibition of endoplasmic reticulum‐derived ROS also alleviated the liver fibrosis. Shin et al stated that exogenous 8‐hydroxydeoxyguanosine ameliorated liver fibrosis by inhibiting the NADPH oxidase signalling.^51^ A plethora of the study suggests that the excessive production of ROS is associated with the pro‐inflammatory signalling pathways activation.

Interestingly, TLR4 is a crucial signalling receptor in the initiation of the inflammatory response.^48^ As mentioned previously, LPS activates the TLR4. It induces inflammation coupled with excessive ROS synthesis. Apart from this, TLR4 influences the NADPH oxidase expression and, in turn, NADPH oxidase activation for ROS production and regulation of inflammation.^52^ The present study shows that the TLR4 gene silencing could attenuate the mitochondrial electron transport complex, and NADPH oxidase generated ROS.

RNA interference is a powerful gene silencing tool. RNAi‐based therapeutics have great potential to target most of the currently undruggable genes and gene products. RNAi therapeutics has been drastically developed over a couple of decades to treat various disorders, such as various cancers, metabolic and genetic diseases and virus infections. The efficacy of several potential RNAi therapeutic agents is being assessed through clinical trials.^53^

In the current study, specific, robust and persistent TLR4 silencing on the HSC surface with further investigations can be translated into RNAi‐based anti‐fibrotic therapy. Current studies have shown that human liver fibrosis is reversible, whereas cirrhosis is irreversible. Therefore, as per the outcomes of the current study, the VitA‐lip‐TLR4‐shRNA treatment has the potential to prevent or reverse the fibrotic process in patients with early cirrhosis. However, its therapeutic efficacy needs further in‐depth investigations in terms of safety and potency. Multiple signal transduction pathways are related to the development of liver fibrosis. Thus, RNAi‐based drugs for liver fibrosis, apart from high targeting efficiency and safety, must be able to target multiple signalling components for an effective clinical outcome.

The outcome of this study suggests that the TLR4‐mediated signalling pathways activation in HSCs plays a crucial role in the fibrogenesis and inflammation in liver fibrosis. This study also demonstrates that the TLR4 shRNA encapsulated within VitA‐coupled liposomes can be a potential therapeutic agent for liver fibrosis treatment.

## CONFLICT OF INTEREST

The authors declare no conflict of interest related to this study.

## AUTHOR CONTRIBUTION

**Yuwei Zhang:** Formal analysis (lead); Methodology (lead); Writing‐original draft (lead). **Yang Li:** Data curation (equal); Formal analysis (supporting). **Tong Mu:** Data curation (supporting); Methodology (supporting). **Tong Nanwei:** Resources (supporting); Writing‐review & editing (supporting). **Ping Cheng:** Conceptualization (lead); Funding acquisition (lead); Investigation (lead); Resources (lead); Writing‐review & editing (lead).

## Supporting information

Tab S1

## Data Availability

The authors confirm that the data supporting the findings of this study are available within the article and its supplementary material.
