# Supplementary material for: Hepatic stellate cells specific liposomes with the Toll‐like receptor 4 shRNA attenuates liver fibrosis
Source: J Cell Mol Med. 2020 Dec 18;25(2):1299–313. doi: 10.1111/jcmm.16209 (PMC7812270; doi:10.1111/jcmm.16209)
Supplement: Supplementary file 1 — Tab S1 [file JCMM-25-1299-s001.docx]

**Supplementary Table 1 The primer nucleotide sequences for qRT-PCR**

| **Gene** | **Primer sequence** |
| --- | --- |
| TLR4 | Sense: 5’-ACCTGGCTGGTTTACACGTC-3’ |
|  | Antisense: 5’-CTGCCAGAGACATTGCAGAA-3’ |
| collagen I | Sense: 5’-TGCCGTGACCTCAAGATGTG-3’ |
|  | Antisense: 5’-CACAAGCGTGCTGTAGGTGA-3’ |
| MMP-2 | Sense: 5’-CCCCGATGCTGATACTGA-3’ |
|  | Antisense: 5’-TGTCCGCCAAATAAACC-3’ |
| MMP-9 | Sense: 5’-CGTCGTGATCCCCACTTACT-3’ |
|  | Antisense: 5’-AACACACAGGGTTTGCCTTC-3’ |
| TIMP-1 | Sense: 5’-CAGAACCGCAGTGAAGAG-3’ |
|  | Antisense: 5’-GGATAGATAAACAGGGAAACA-3’ |
| TIMP-2 | Sense: 5’-TTCCGGGAATGACATCTATGG-3’ |
|  | Antisense: 5’-GGGCCGTGTAGATAAACTCGAT-3’ |
| p22phox | Sense: 5’-TGGTATTTCGGCGCCTACTC-3’ |
|  | Antisense: 5’-CCGACAACCATCGCTCCAT-3’ |
| gp91phox | Sense: 5’-CCCTCCCTGTCTAGGTAATGC-3’ |
|  | Antisense: 5’-GCATTTGCCTTCGGTGATGT-3’ |
| p40phox | Sense: 5’-GTGGCTGAGAAGACGTGTGA-3’ |
|  | Antisense: 5’-TTCCTGAAGCTTGGGGTGTC-3’ |
| p47phox | Sense: 5’-CTGGAGGGCAGAGACAATCCA-3’ |
|  | Antisense: 5’-CTGCTTCTCACACAGCGGA-3’ |
| p67phox | Sense: 5’-AGTGTAGTGGCTGCACATCT-3’ |
|  | Antisense: 5’-GGGATGTAACCCAGGCTTGT-3’ |
| Rac-1 | Sense: 5’-GCCTGCTCATCAGTTACACG-3’ |
|  | Antisense: 5’-TACCACTTTGCACGGACATT-3’ |
| GAPDH | Sense: 5’-ACTCCACTCACGGCAAATTC-3' |
|  | Antisense: 5’-TCTCCATGGTGGTGAAGACA-3' |
